# Harmonising topographic & remotely sensed datasets, a reference dataset for shoreline and beach change analysis

**DOI:** 10.1038/s41597-019-0044-3

**Published:** 2019-04-26

**Authors:** James A. Pollard, Susan M. Brooks, Tom Spencer

**Affiliations:** 1 Coastal Research Unit, Department of Geography, University of Cambridge, Downing Place, Cambridge, CB2 3EN UK; 2 Department of Geography, Birkbeck, University of London, Malet Street, London, WC1E 7HX UK

**Keywords:** Natural hazards, Physical oceanography

## Abstract

This paper presents a novel reference dataset for North Norfolk, UK, that demonstrates the value of harmonising coastal field-based topographic and remotely sensed datasets at local scales. It is hoped that this reference dataset and the associated methodologies will facilitate the use of topographic and remotely sensed coastal datasets, as demonstrated here using open-access UK Environment Agency datasets. Two core methodologies, used to generate the novel reference dataset, are presented. Firstly, we establish a robust approach to extracting shorelines from vertical aerial photography, validated against LiDAR (Light Detection and Ranging) and coastal topography surveys. Secondly, we present a standard methodology for quantifying sediment volume change from spatially continuous LiDAR elevation datasets. As coastal systems are monitored at greater spatial resolution and temporal frequency there is an unprecedented opportunity to determine how and why coastal systems have changed in the past with a view to informing future forecasting. With revelation of trends that suggest increasing coastal risk, coastal change research is needed to inform the management and protection of coasts.

## Abstract (summary)

**Table:** 

Design Type(s)	time series design • modeling and simulation objective
Measurement Type(s)	shoreline
Technology Type(s)	computational modeling technique
Factor Type(s)	position • elevation
Sample Characteristic(s)	Scolt Head Island • sea coast

Machine-accessible metadata file describing the reported data (ISA-Tab format)

## Background & Summary

It is estimated that low-lying coastal environments comprise 2% of global land area whilst supporting >10% of the global population^1^. Furthermore, coastal environments are associated with a high concentration of critical infrastructure^2^ and valuable ecosystems^3,4^. Technological advance is revolutionising our ability to collect datasets relevant to the study of coastal systems^5^ and their management^6,7^. This includes improvements in the temporal frequency and resolution of datasets such as aerial imagery which have been collected for decades. It also encompasses improved spatial coverage of surveying techniques such as LiDAR (Light Detection and Ranging) which have historically been limited to reactive event-response surveys. Alongside these more standard data sources, satellites offer the possibility of weekly image captures with capabilities to provide multi-spectral datasets globally. Though extensive geoscience Earth Observation satellite programmes have been operating since the 1970s it is only since the advent of analysis platforms such as Google Earth Engine^8^ that global scale coastal analyses have become feasible^9,10^. Deployed on a local basis, and requiring substantially less capital investment, Unmanned Automated Vehicles (UAVs) present a relatively low-cost approach to local coastal monitoring with data quality improving as payload limits and vehicle stability are increased. As an approach, much of the value in remote sensing derives from the ability to collect data in regions that are inaccessible, costly, or dangerous to measure using standard field survey techniques.

Yet, simply continuing to generate data is insufficient if improved understanding of coastal dynamics and effective coastal management is to be achieved. To convert this data into valuable research and policy output, standard approaches to data organisation, harmonisation and analysis are required. Existing efforts to collate coastal data include: EUROSION (erosion rates on European coasts)^11^, DIVA (Dynamic Interactive Vulnerability Assessment, global scale wetland change)^12^, LOICZ (Land-Ocean Interactions in the Coastal Zone)^13^, SurgeWatch (storm surge characteristics around the UK)^14^, and the Mediterranean Coastal Database (sea level rise and associated hazards in the Mediterranean)^15^. These efforts have generated extensive databases with the potential for further research and policy application. In addition to collated coastal datasets, there is a need for consistent approaches towards pre-processing of the data before being deposited for others to access. Yet, there is little by the way of ‘best practice’ for synthesising and harmonising diverse coastal datasets. The lack of standard methodologies among the scientific/academic community imposes limitations on the outputs and interpretation of potentially highly valuable data:

- Limited comparability to similar studies conducted elsewhere.
- Questionable consistency in the quantification of uncertainties.
- Difficulties in establishing priority areas (for either research or intervention) within a national or local context.

These limitations represent serious impediments to the potential for coastal research to usefully inform coastal risk management. Overcoming these shortcomings ought to be a central aim given endorsements^16^ (and subsequent enshrinement in policy objectives^17^) of the need for holistic thinking, both in the assessment of risks and subsequent mitigation or adaptation interventions. This paper addresses the need for consistency in the critical conversion from raw data to value through presenting a novel reference dataset and associated core methodological techniques (each comprised of a set of procedures) which shed light on coastal system functioning. It is critical that the reference dataset and the methodologies used to generate it are considered jointly. Firstly, we describe a robust methodology for shoreline change analysis using vertical aerial photography, validated against LiDAR elevation models and cross-shore topography surveys. Secondly, we present a technique for determining sediment budgets using temporally separated elevation datasets from which volumetric data can be obtained. These methodologies facilitate use of both recently collected datasets and (where available) reanalysis of existing data through a novel methodological lens to generate a novel reference dataset

**Fig. 1:**
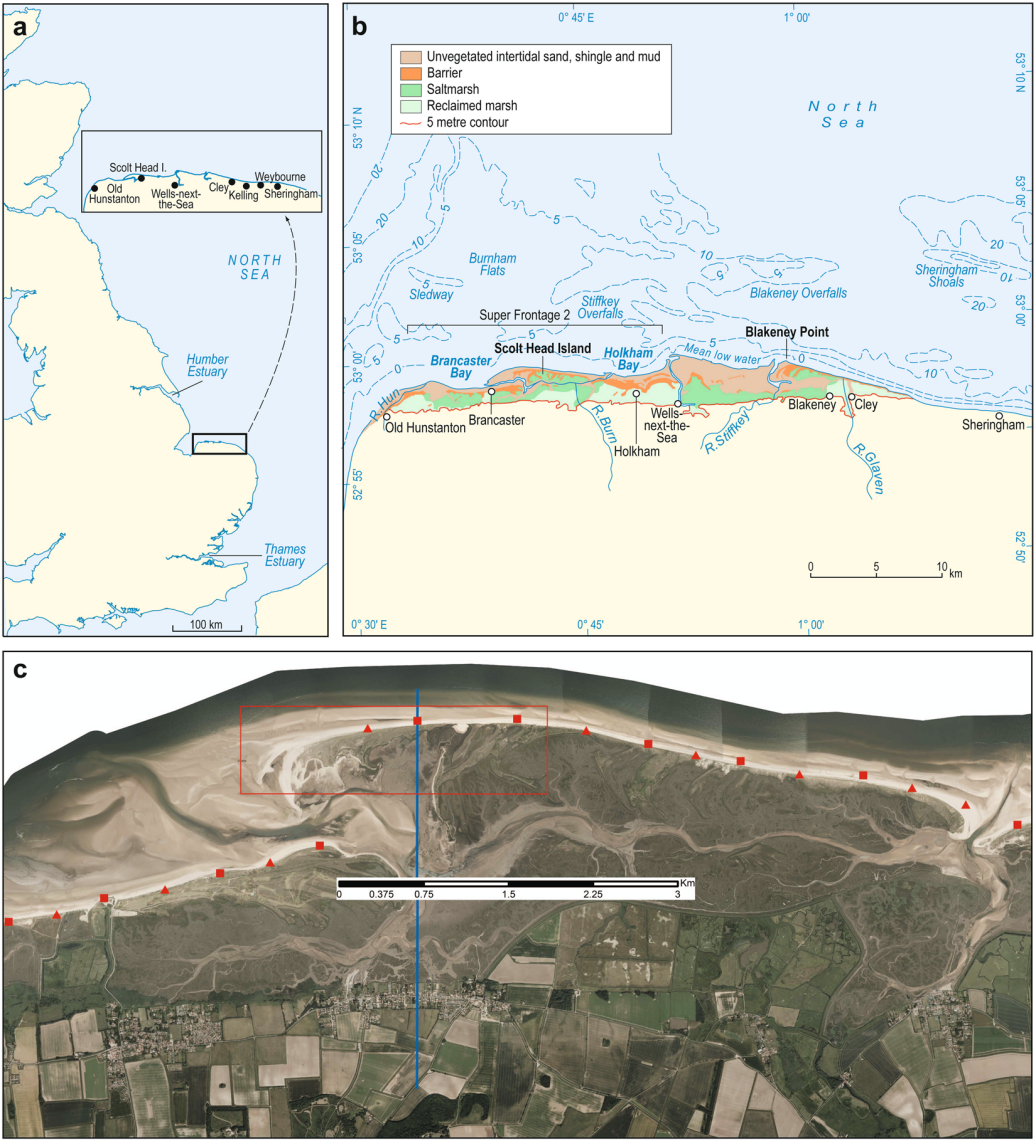
Site Map, Scolt Head Island, North Norfolk Coast. (**a**) General setting of the North Norfolk Coast. (**b**) Detailed locations of the habitats and places along the North Norfolk coast showing the location of Scolt Head Island; and (**c**) 2013 aerial imagery of Scolt Head Island showing the locations of the EA cross-shore topography surveys (triangles are newer profile locations (since 2011) and squares are the original (since 1992) profile locations). Also shown are the extent of the detailed LiDAR analysis of the changing western end of Scolt Head Island (red polygon) and one example of a cross-shore topography survey (N014) with elevations available at every cross-shore point (blue vertical line).

**Fig. 2:**
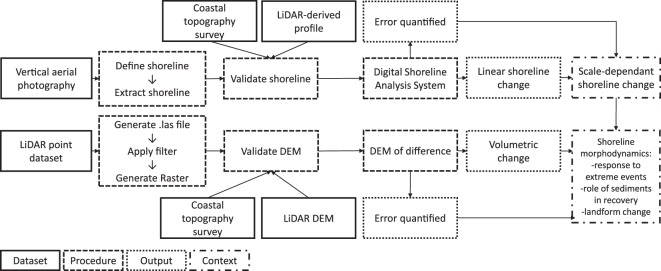
Summary of dataset harmonisation for coastal change research. Abbreviations: DEM (Digital Elevation Model); LiDAR (Light Detection and Ranging). Elaboration of the vertical aerial photography procedures are found in Section 2: Shoreline Change. Elaboration of the LiDAR point dataset procedures are found in Section 3: Elevation Change.

Three primary datasets are handled: vertical aerial photography, LiDAR and coastal topography surveys (Table 1). These are standard data types that are already available in various forms in coastal locations globally. In cases where these datasets are not available, the robust validation techniques presented here cannot be applied and thus a higher degree of uncertainty will likely accompany the resulting analysis in the absence of alternative validation procedures. The novel dataset and associated methodologies presented here are validated on a local scale through an application to storm impact assessment at Scolt Head Island, a sandy/gravel barrier system located on the UK’s North Norfolk coast (Fig. 1). In this dataset production and validation study, all primary datasets were collected by the UK Environment Agency (EA) and are openly available. We also include field data collected in the aftermath of the 5 December 2013 UK east coast storm surge^18^. In this paper, we emphasise a logical stepwise approach to method design and execution that is applicable to other national (and local) data inventories. The key harmonisation of the datasets and the logical steps towards subsequent analysis are presented in Fig. 2.

**Table 1:** Coastal datasets for harmonisation.

Dataset	Originator	Access	Temporal range	Temporal resolution	Spatial resolution	Type	Format
Vertical aerial photography	Environment Agency	http://environment.data.gov.uk; https://www.channelcoast.org/	2008-present 2001-present	Annual	10–25 cm	Remotely-sensed	TIFF
LiDAR DSM, DTM & point clouds	Environment Agency	http://environment.data.gov.uk; https://www.channelcoast.org/	1999-present 2004-present	Annual	25–200 cm	Remotely-sensed	TIFF
Coastal topography surveys	Environment Agency	https://www.channelcoast.org/	2000-present	Biannual	0.2–1 km	Field survey	Text file

## Methods

### Data acquisition

This paper focuses on three datasets capable of capturing shoreline change: vertical aerial photography, LiDAR (Light Detection and Ranging) DEMs (Digital Elevation Model) and coastal topographic surveys. The ease of data acquisition and computational processing for use within a Geographical Information System (GIS) varies between them. Throughout this paper, we refer to primary datasets collected by the UK Environment Agency (EA), though the associated procedures are generic in nature. We provide a detailed description based upon the EA datasets and the procedures we develop can be applied to similar datasets from varied sources.

Aerial photography has been collected annually by the EA every boreal summer (to ensure seasonal comparability) since 1992 between the estuaries of the Humber and the Thames on the UK east coast. Images back to 2001 are available (see Table 1). Two high level classifications are vertical and oblique imagery. This paper deals exclusively with vertical imagery, though acknowledges the value of oblique imagery particularly through application of photogrammetry techniques^19^. Single band (greyscale) images were collected from 1992–2000 for the most comprehensively monitored regions of the UK, RGB (red-green-blue) images are available from 2001–2010 and RGBI (red-green-blue-infrared) images were collected from 2011–2016. The EA is currently in the process of uploading older photography to the open access data portal (http://environment.data.gov.uk/) though availability varies markedly between different localities. Flight dates for vertical aerial photography is contained in the photograph file names for later series and further metadata is available from the EA^20^.

Light Detection and Ranging (LiDAR) consists of elevation data collected by airborne laser scanning technology. LiDAR has been collected regularly in England and Wales since 2008 but as early as 1999 in certain localities. The EA provides various ‘flavours’ of LiDAR data. Digital elevation modes (DEMs) containing mosaics of the most recent survey at a single spatial resolution (typically 1 m or 2 m) are a common format. These DEMs may be digital surface models (DSMs) or digital terrain models (DTMs), with the distinction between them being the inclusion (DSM) or removal (DTM) of surface vegetation and other visible structures. Finally, in some locations LiDAR point clouds are the only format provided, but this enables the user to impose their own choice of interpolation technique and filter to generate an elevation model. Remotely sensed datasets and associated metadata are increasingly available in various formats from the Channel Coastal Observatory portal (http://www.channelcoast.org/).

Coastal topography surveys are cross-shore transects collected on the ground on a biannual timeframe, again for the most comprehensively monitored regions dating back to 1992, initially at 1 km alongshore spacing but with more recent higher spatial densification in areas of particular interest where the coast is undergoing rapid change. Hence two coastal topography survey series exist, the 1 km spaced profiles, carried out since 1992 and interspersed profiles, carried out since 2011. The biannual survey schedule ensures that one summer and winter profile is captured per year, providing a ‘before and after’ dataset which is particularly useful for assessing coastal change during energetic winter months. They provide a vital corroborating dataset for shoreline change analysis when assessed alongside vertical aerial photography and LiDAR. The cross-shore profiles are available from present to 2000 on the Channel Coastal Observatory portal (http://www.channelcoast.org/) where they can be freely downloaded. Using these three datasets we develop and outline a series of procedures in the following section that ultimately generate a detailed quantitative assessment of coastal change. A summary of these procedures can be found in Table 2.

**Table 2:** Summary of datasets and procedures. Detailed descriptions of each protocol can be found in the Usage Notes.

	Vertical aerial photography^30^	LiDAR DEM^31^	LiDAR point cloud^34^	Cross-shore profiles^34^
Protocol 1	Mosaic AER_YYYY_mos	Import DEM LID_YYYY_raster	Convert to.las LID_YYYY_LAS	Import DEMs LID_YYYY_raster
Protocol 2	Clip AER_YYYY_clip	Create line shapefile LID_N014_line	Convert to raster LID_YYYY_raster	Create shapefile of large area LID_YYYY_YY_roi
Protocol 3	Geo-reference AER_YYYY_geo	Create point shapefile LID_YYYY_N014s _prof	Contour raster MHWS_YYYY	Minus LID_YYYY_YY_dod
Protocol 4	Greyscale function AER_YYYY_gr	Create points LID_YYYY_N014s _prof		Create shapefile of small area LID_N014_roi
Protocol 5	Convolution function AER_YYYY_sobhv	Add surface information LID_YYYY_N014s _prof		Extract area LID_YYYY_N014 _raster
Protocol 6	Convert to bitonal AER_YYYY_bit25			Minus LID_YYYY_YY _N014_dod
Protocol 7	Vectorise AER_YYYY_vectors			Collate profiles for validation LID_YYYY _westSHI_prof
Protocol 8	Extract shoreline AER_YYYY_sline			
Protocol 9	Conduct shoreline analysis AER_YYYY_YY_sce			

### Shoreline change

#### Procedures

At first glance, the ‘shoreline’ can be simply defined as the ‘point of intersection between land and sea’^21^. However, this definition fails to acknowledge that this point of intersection will reflect processes that are specific to that moment in time and that position in space. The processes acting at any given time and space combination are not necessarily constant, nor representative of the ‘average’ shoreline position^22^. As a result, each measurement of the land-sea interface may differ drastically from the next, not due to any directional change in geomorphological processes, but purely because of spatiotemporal variation occurring at a greater rate than the frequency of sampling^23^. Taken together, vertical aerial photography, LiDAR and coastal topography surveys each capture subtly different information and thus can be harmonised to obtain a robust shoreline proxy that overcomes some of these limitations.

##### Shoreline definition

On vertical aerial photographs it is necessary to utilise shoreline proxies that are visually discernible coastal features^24^. There are numerous examples, from transient features such as drift lines and wet/dry lines, to longer-lived forms, such as beach berms, vegetation lines and cliff edges. The choice of shoreline proxy has been shown to influence the shoreline change patterns detected^25^. Drift lines and wet/dry lines are the most commonly found within the shoreline change analysis literature^22^. They indicate the position of the last High Water Line (HWL) from the previous high tide. The use of the HWL as a shoreline proxy has be widely critiqued because it is not always obvious, may appear as a zone rather than a distinct line, and can be confused with other shore-parallel lines. It has also been suggested that the HWL may reflect conditions several days prior to the time of survey rather than merely the last high tide^25–27^. We also have to question the degree to which the last HWL position reflects the average shoreline position, given seasonal and secular tidal fluctuations as well as wave runup. Long-term (historic centennial scale) shoreline change requires matching contemporary shorelines with those on historic maps where the wet-dry line is not evident.

Acknowledging these shortcomings, it has been suggested that features higher up the beach profile ought to be used owing to their greater longevity and resistance to such short-term fluctuations^28^. One drawback of selecting a more permanent feature is that such features may fail to respond to the processes responsible for shoreline change elsewhere across the beach profile. This paper suggests that the use of a vegetation line provides an appropriate balance between responsiveness to beach dynamics and detectability from remotely sensed datasets. The use of a vegetation line introduces its own limitations, the foremost being that the beach-vegetation transition does not always form a quasi-continuous line. Rather, the transition may be characterised by patchiness due to the presence of pioneer dune formation or local vegetation dieback. A discontinuous vegetation line occurs at certain locations on Scolt Head Island, for example at the inlets either side of the barrier where frequent inundation and sediment mobility preclude extensive vegetation development. In such locations, the variability in the vegetation line introduces ‘variability error’ which is captured during the validation procedure presented below. It is important to be aware that this will result in a spatially variable error along the shoreline extracted. Of course, shoreline proxy selection will remain a subjective decision, dependant to some extent on location-specific characteristics. For the reasons elaborated above, where a vegetation line is present, and reasonably continuous, it provides an appropriate choice of shoreline proxy. In the absence of a vegetation line, for example, on hyperarid coasts or where the beach is backed by an artificial coastal defence structure, the HWL might be selected as an alternative shoreline proxy. In the case of a water-line derived proxy, new opportunities for validation may arise through comparison with tide gauge datasets, for example^29^. Given the diverse nature of coastal systems, a discussion of the reason for selecting a particular proxy, and a discussion of how this decision impacts the results obtained, is crucial in any rigorous assessment.

##### Shoreline extraction

As detailed above, shoreline definition is especially important for accurately positioning the shoreline when dealing with vertical aerial photography. Vertical aerial photography downloaded from the EA data portal comes georeferenced (ortho-rectified) ‘using simultaneous LiDAR and GPS to a high spatial accuracy’^20^. In the validation section below, we include a procedure for quantifying the relative error between successive vertical aerial photographs. This is achieved using the later photograph as the reference for the earlier one (Data workflow 1). Having obtained or generated a georeferenced image, the shoreline extraction procedure can begin (Data workflow 2). Here, we detail the extraction of a vegetation-based shoreline proxy. This procedure would need to be modified for extraction of alternative shoreline proxies. During the georeferencing procedure individual photograph tiles will have been merged into a mosaic. The merging of numerous photograph tiles tends to create a large file which can result in long processing times. It is therefore recommended to ‘clip’ the aerial photograph to the broad area of interest. When dealing with colour images, conversion to greyscale is necessary. To increase the visual contrast of the shoreline a series of edge detection algorithms are applied. Numerous algorithms (including Laplacian and segmentation mean shift) were trialled. The most effective edge detection algorithm in this case was found to be the Sobel convolution function, which can be applied in a vertical or horizontal plane. Given that shorelines do not necessarily follow an exclusively horizontal or vertical direction, it is most effective to perform each of the convolutions independently and then combine them using a square root function. This removes the preference for exclusively horizontal or vertical shoreline extraction, which emerged when using either horizontal or vertical Sobel function in isolation. The vertical aerial photograph is then converted into a bitonal image. Here, threshold selection is important and must be decided (in some cases iteratively through trial and error) to ensure that the desired shoreline is emphasised relative to its surroundings. Vectorisation is applied to the bitonal image to automatically extract the shoreline in shapefile format. For further details on the vectorisation settings used, see Data workflow 2. A cleaning step is required to remove irrelevant vectors and merge disparate shoreline sections. This step may also require some manual shoreline tracing in areas where shorelines have not been adequately vectorised^30^ (Table 2). The final shoreline vector should be validated against cross-shore topography surveys, as explained in the validation section below^31^ (Table 2).

##### Shoreline change analysis

Once the shorelines have been extracted, and the associated errors quantified (see validation section below), accurate shoreline change analysis can be performed. Various open source software for shoreline change detection exist. One example that is becoming widely used among coastal practitioners is the ArcMap plugin Digital Shoreline Analysis System (DSAS) v4.4^32^. This provides a densified dataset by casting transects at more frequent intervals than would be possible to measure in the field, and the transect spacing can be user-defined. Detailed instructions for installation and use can be found online (https://woodshole.er.usgs.gov). Another open-access alternative is to use the R package AMBUR (Analysing Moving Boundaries Using R, http://ambur.r-forge.r-project.org/). In addition to the fact that AMBUR is open access, it also has capabilities for dealing with curved shorelines^33^. Instructions for using the AMBUR package are provided on the associated website.

Relying on field-based coastal topography surveys alone for shoreline change detection masks alongshore patterns of shoreline change, especially where datapoints are only available at 1 km alongshore spacing. Using the profiles to validate the vertical aerial images (see validation section below) and in turn extracting a quasi-complete shoreline, provides a more detailed picture of coastal margin change. The recent availability of LiDAR datasets provides a more comprehensive set of elevation data to detect patterns and magnitudes of alongshore variability in the shoreline response to forcing. Crucially through overlaying successive LiDAR datasets, it is possible to determine sediment volume change both alongshore and cross-shore. We now outline a standard method for doing this that includes routines to interface and use the different formats in which LiDAR data are supplied.

### Elevation change assessment

#### Procedures

To quantify the magnitude and spatial distribution of sediment volume change, it is necessary to create a DEM of difference (DoD) between the years of interest for a specific defined area of the coastal zone. Here, a method that integrates point cloud and pre-processed ASCII LiDAR datasets is presented, although it is equally possible to rely on just one of these data formats^34^ (Table 2).

##### Point cloud data

**Fig. 3:**
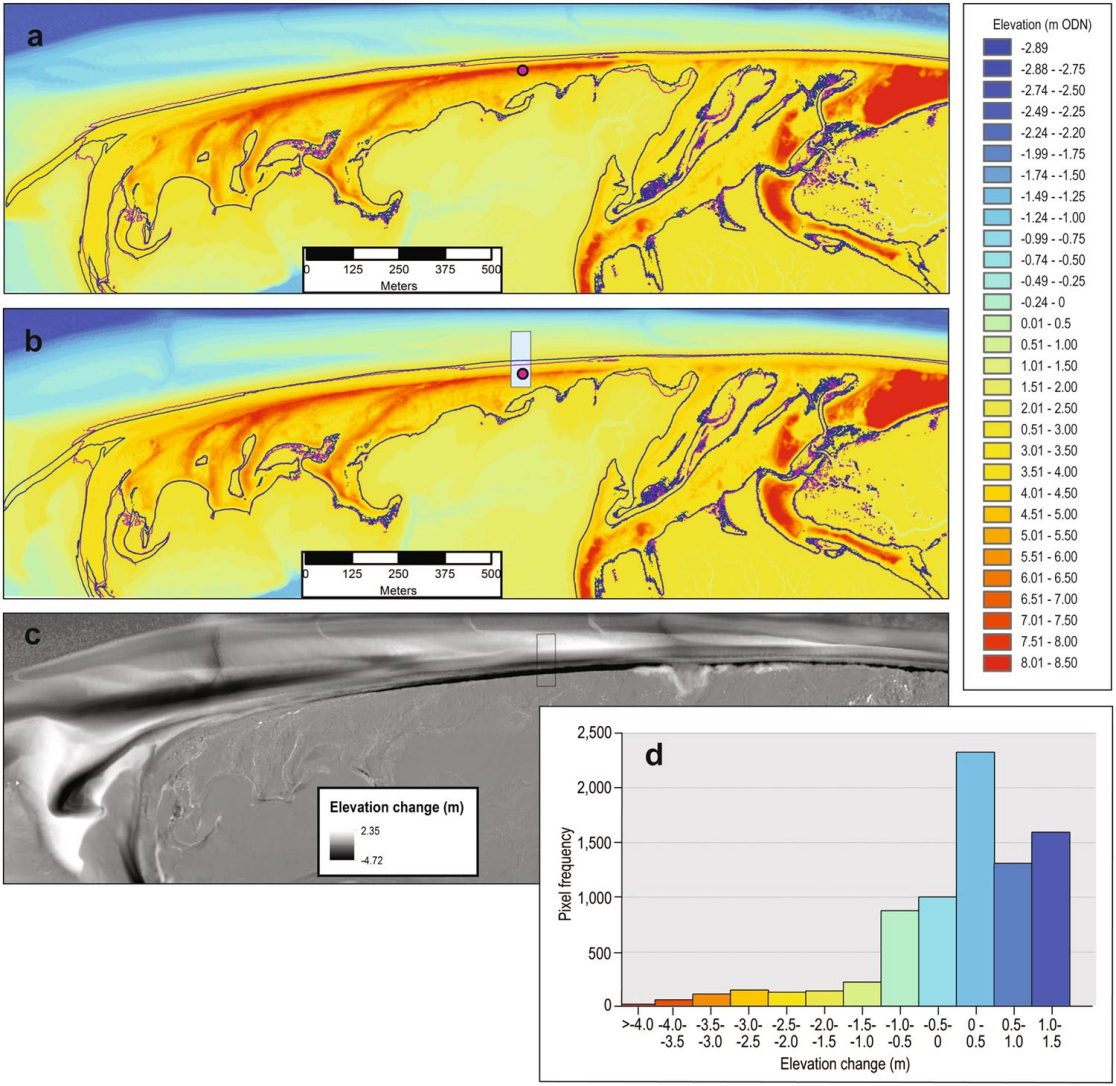
DEM of difference at Scolt Head Island between two successive years. A DEM derived from (**a**) the 2013 LiDAR point cloud (23^rd^ January 2013); (**b**) the 2014 ASCII files for the western end of Scolt Head Island (28^th^ February 2014). Extraction of identical spatial coverage is achieved with the Extract by Mask ArcMap tool. Green lines locate MHWS for 28^th^ January 2013 and purple lines locate MHWS for 28^th^ February 2014. Island (cross-shore survey N014 is shown for reference). (**c**) DEM of Difference (DoD) from 28^th^ January 2013 to 28^th^ February 2014 for identical spatial coverage at the western end of Scolt Head (an area around N014 experiencing barrier lowering and retreat is shown as a black bounded box). (**d**) Elevation change distribution from the DoD for the area around N014.

It is possible to generate DEMs from point cloud datasets using the open source ‘laszip’ code for conversion from .laz to .las file formats (https://laszip.org/; Data workflow 4). It is necessary to apply a filter to generate the DTM, so that surface vegetation and other structural features are excluded from the DEM. The filter should include only the last returns or points classified as ground to generate a bare earth surface. The .las dataset should then be converted into raster format so that it can be easily manipulated and compared with other DEMs from other time periods for the same area of interest. When converting to raster format the user must specify an average cell resolution; following a series of trails using cell resolutions of 0.25 m, 0.5 m, 1 m and 2 m which comply with the ASCII format DEMs supplied via the open-access data portal (http://environment.data.gov.uk/), we proceeded with a resolution of 1 m. The trials involved performing a linear regression analysis on each of the rasters generated at the different cell resolutions tested against ground truth data. The average elevation difference (taken after converting all deviations to positive values) between the 2 m raster and the ground truth data was 0.135 m, for the 1 m raster the average difference was 0.109 m, while for the 0.5 m and 0.25 m rasters the average differences were 0.108. These differences may seem small but they are magnified when extrapolated over a wide area to make volume change calculations. It was therefore considered appropriate to use a 1 m cell resolution, as there was only limited further accuracy achievable by resolving to a smaller cell size. A 1 m cell size is also the most readily available product available for other years when data are supplied in ASCII format. The next decision was in the choice of interpolation method to use, including how to assign values to the cells, as well as how to treat cells with zero values. The method chosen to assign values to cells was to take the average value of all the points that fell within that cell. The options we explored for void treatment included assigning no data, using the average value of all adjacent cells to fill the void, or performing either linear or natural neighbour interpolation across the void to determine its value. However, as the average point spacing in the .las dataset was 0.74 m and the cell resolution was 1 m very few voids needed to be interpolated. We found negligible differences in the rasters generated using each method. On comparing each to a set of ground survey control points, the Natural Neighbour Interpolation method was found to be statistically more robust than other methods. Once generated, the DEM should be displayed in a fashion appropriate to the vertical elevation range (Fig. 3a,b). To ensure consistent comparison between DEMs, it is necessary to define an area of interest such that the DEMs to be compared cover the same area. This can easily be achieved by creating a polygon shapefile to form a mask of the area, and then extracting that area from each raster. Using the extracted raster area as an input, contours of equal elevation can be constructed for any user-specified value. In this application, adding Mean Sea Level (MSL) and Mean High Water Springs (MHWS) defines the zone of active marine activity under normal conditions, hence the region where you would expect to find regular change in elevation. Figure 3a,b show the contour for January 2013 and February 2014 for MHWS, highlighting their changing positions over this time period. The difference in contour position suggests flattening of the upper intertidal beach profile and erosion of the supratidal dunes during the energetic winter of 2013–14^35^. In the DoD (Fig. 3c) clear patterns of change are evident between January 2013 and February 2014; these patterns are discussed further below. The elevation change histogram for this period is shown as Fig. 3d, providing a means of displaying the overall elevation change distribution following which further statistical tests of difference between rasters for different periods can be performed.

##### DEMs of difference

To create a DEM of difference, it is necessary to have two LiDAR DEMs in the same vertical units and horizontal resolution, from different time periods (Data workflow 5). In this example we use the LiDAR point cloud to generate a 1 m resolution DEM for January 2013 to compare against the ASCII DEM from February 2014 which is publicly available as a derived DTM product. The LiDAR point cloud was filtered and used to generate a 1 m horizontal resolution raster and then vertical units were converted into m ODN (Ordnance Datum Newlyn, where 0.0 m ODN approximates to UK mean sea level; as the point cloud is supplied in mm units). Both rasters were symbolised to the same elevation bins to ease visualisation (Fig. 3a,b).

For accurate comparison of topographic change between January 2013 and February 2014 we use identically-sized areas, by creating a polygon shapefile and then extracting by mask the rasters corresponding to these two time periods. The Digital Elevation Model (DEM) of Difference (DoD) was then generated by subtracting the 2013 elevations raster from the 2014 elevations raster (Fig. 3c).

The histogram showing the distribution of elevation change is shown in Fig. 3d. As the DoD covers a substantial area of back barrier marsh as well as the barrier itself, there is strong clustering of pixel change around zero. The distribution contains a total of 111 098 pixels, with a mean of 0.05 m and a standard deviation of 0.38 m. The minimum and maximum values are −4.7 and 2.3 m respectively, and it is clear from the DoD (Fig. 3c) where the maximum surface elevation losses (barrier erosion) and maximum gains (beach development and deposition around the pre-existing overwash fan) have taken place.

Thus, the shapefiles, LiDAR and DoDs can be used together to elucidate barrier dynamics and responses to storms for any point along the barrier. For example, a shapefile set up for the area surrounding EA cross-shore survey N014 (running from MSL to 150 m landward and 50 m alongshore (Fig. 3b) can be used as a template for extracting the DoD of this smaller area. The visualisation of change is more focussed when a smaller area is taken. The histogram of change (as exemplified in Fig. 3d for the whole area) allows comparison between different parts of the barrier, possibly with differing exposure, as well as for different regions within the tidal frame (e.g.: upper or lower intertidal beach, or supratidal beach-dune foot) and for different years where LiDAR are available.

## Data Records

The novel reference dataset presented here is openly accessible from PANGAEA, a data publisher for earth and environmental science, where it is organised into four data entries. Each data entry contains a file for each methodological protocol (Table 2). The first data entry^30^ comprises files for each procedure involved in the processing of vertical aerial photographs. This consists of six raster files (representing protocols one to six) and three shapefiles (representing protocols seven to nine). The second data entry^31^ comprises files for each procedure involved in the processing of LiDAR DEMs to extract cross-shore topographic profiles. This consists of the initial DEM raster file and four shapefiles representing subsequent procedures. The third data entry^34^ concerns the elevation change assessment procedures. Three files are included to demonstrate the conversion of a .las dataset to a raster dataset and subsequent delineation of a spatially consistent area for the DoD calculation. Four raster files and three shapefiles are presented to demonstrate the calculation of a DoD from two raster datasets and subsequent validation against cross-shore profiles^34^. The fourth and final data entry^36^ contains a Microsoft Excel spreadsheet (XLSX) containing shoreline error metadata for the shorelines extracted from the vertical aerial photographs.

## Technical Validation

### Shoreline change

Throughout shoreline definition and extraction procedures, various sources of error are introduced that must be acknowledged in the final shoreline. Acknowledgement of the error introduced is critical to ensure that subsequent shoreline change analysis is detecting genuine change rather than noise introduced by the procedures employed to extract shorelines. In vertical aerial photography, error arises because of differences between the ‘image space’ of the photograph itself and the ‘object space’ that is being photographed^37^. Distortions are relatively more important in smaller scale photographs, where ground relief is greater, and where photographs are taken at lower altitude^37^.

#### RMST

Equation 5 is a formula developed for error analysis of historical maps^38^. It can be applied to vertical aerial photography.

1 $$RMST=\sqrt{RMS{S}^{2}+RMS{I}^{2}+RMS{V}^{2}}$$

RMST = root-mean-square total error

RMSS = root-mean-square source error

RMSI = root-mean-square interpretation error

RMSV = root-mean-square variability error

RSMT shoreline error was calculated for SHI between 2013–2014^36^. The relative contribution from each component of RMST will vary depending on the nature of datasets employed. In this example, the greatest error term is introduced by RMSI, quantified using cross-shore topography surveys and LiDAR profiles. The use of LiDAR-derived profiles, sampled at 1 m cross-shore, reduces the RMSI by an average of 4.26 m by comparison to using cross-shore topography surveys alone. This demonstrates the value of utilising high-resolution elevation datasets when available.

#### RMSS

RMSS is the accuracy of a point compared to its actual location on the ground. Quantifying RMSS for vertical aerial photography is a relatively simple procedure, in which selected fixed points are noted and compared to the same points as recorded on modern georeferenced imagery. At least five fixed points are selected per mosaic, and more if possible. Every effort is made to select reliable fixed points that are close to the shoreline, but we recognise that this may not always be possible because of the dynamic nature of the coastal zone. It is particularly problematic establishing sufficient seaward fixed points. When dealing with vertical aerial photography, higher resolution images are easier to georeference with high accuracy, as are colour images given that they allow features to be more easily distinguished.

#### RMSI

**Fig. 4:**
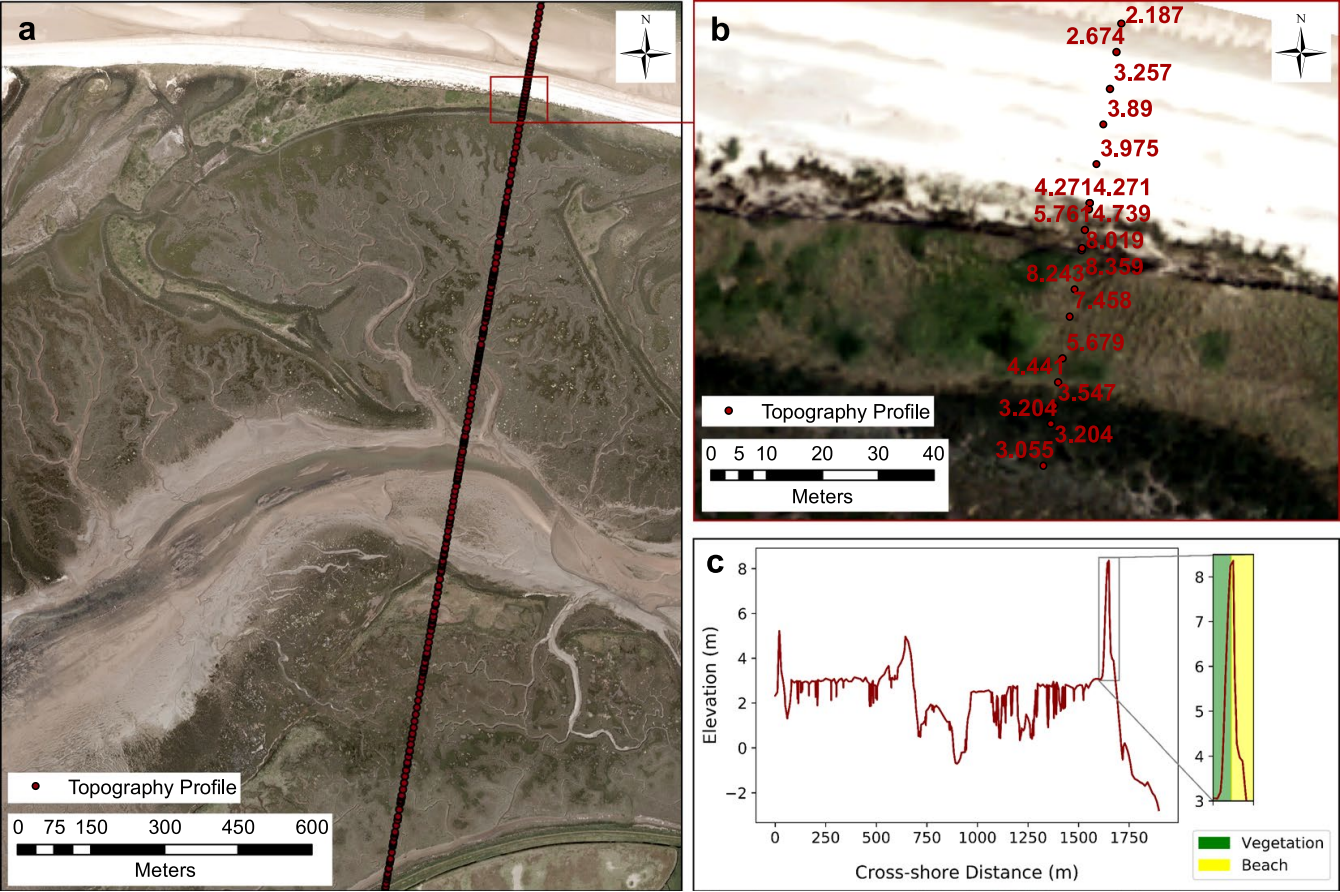
Validation of the vegetation-based shoreline proxy using a coastal topography survey. (**a**) Environment Agency cross-shore topography survey. (**b**) Cross-shore topography survey labelled with elevation (m ODN). (**c**) Cross-shore distance plotted against elevation to demonstrate coincidence between elevation change and vegetation shoreline.

RMSI quantifies the error introduced by the digitizer in their interpretation of where the shoreline proxy lies. Field surveys provide a triangulation of techniques which allows for greater confidence in the extraction of shoreline position, as detailed in the ‘shoreline extraction’ section above^29,35^. Coastal topography surveys introduce a small degree of vertical error. The UK Environment Agency uses a Leica Global Navigation Satellite System (GNSS) which limits 3D coordinate errors to <20 mm (http://www.channelcoast.org/). Taking advantage of this high accuracy elevation dataset ensures that the correct shoreline is extracted, minimizing the occurrence of error. The coastal topography survey closest in date to the vertical aerial photograph can be displayed in GIS software alongside the vertical aerial photograph (Fig. 4a,b). Since the coastal topography survey is overlaid on the photograph, it should be possible to identify a break in slope on the coastal topography survey that corresponds with the vegetation line on the photograph (Fig. 4c).

**Fig. 5:**
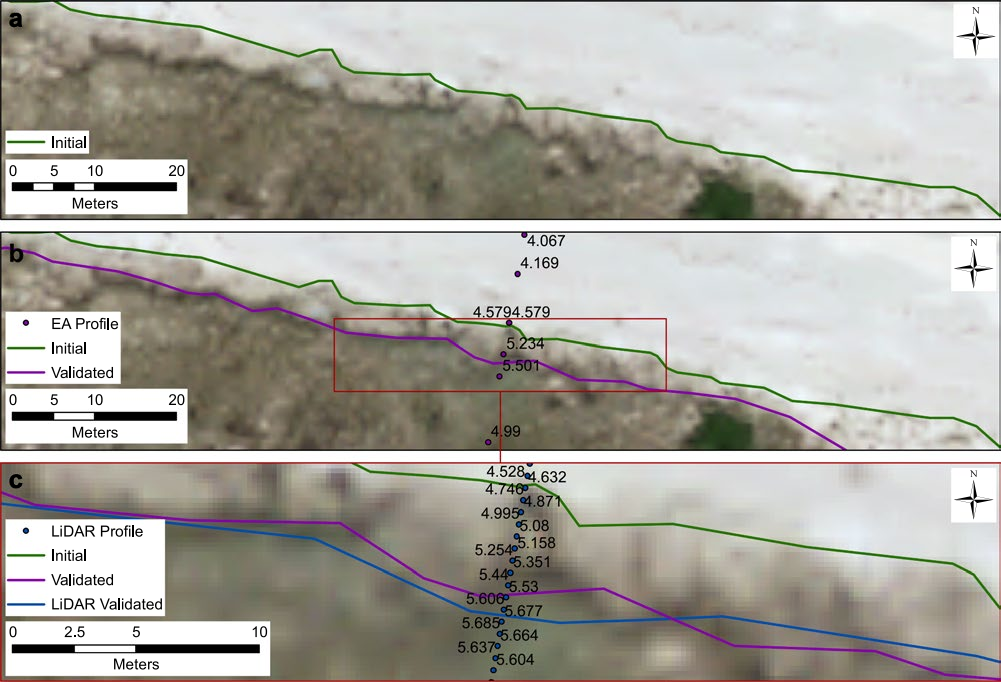
Quantifying shoreline interpretation error through elevation-based validation. (**a**) Initial shoreline based on visual assessment of vegetation line; (**b**) amended shoreline using EA profile for validation; (**c**) final shoreline using LiDAR derived profile for validation.

Initially, and in the absence of appropriate elevation data, a vegetation line may be extracted based purely on visual inspection (Fig. 5a). Given that the vegetation proxy is represented by a point of inflection on the cross-shore topography survey, it is possible to quantify the maximum possible interpretation error, which is equivalent to the distance between the two profile points either side of the maximum point value (Fig. 5b). This is an upper estimate of the error associated with the shoreline. If LiDAR elevation data are also available at a higher resolution (as is typically the case) than the cross-shore topography survey, then the shoreline position can be further refined (Fig. 5c). In this case, refinement of the shoreline position is facilitated by UK Environment Agency LiDAR which has a vertical accuracy of <150 mm (http://www.channelcoast.org/).

The LiDAR data should be pre-processed from a point cloud as described above or the product downloaded directly from one of the open-access data portals. In both cases, the LiDAR must be mosaiced and converted to a vertical datum consistent with the topography profiles. For the datasets described here, we use m ODN. Topography profiles are extracted from the LiDAR datasets in the same location as the coastal topography survey (Data workflow 5). This is achieved by creating a point shapefile at the chosen transect location and mapping the LiDAR elevation data onto it.

If the profile is overlain on the vertical aerial photograph, we can apply the same validation methodology as with the coastal topography survey. However, this time, the interpretation error can be consistently limited to 1 m along the entire shoreline (0.5 m either side of the highest point at the edge of the visible vegetation). This represents a significant improvement over using topography survey with uneven spacing of point measurements (compare Fig. 5b,c). Given that LiDAR is not available at all coastal sites, two RMSI values are provided in the shoreline error metadata^36^. One based on coastal topography surveys and the other based on interpolated LiDAR derived topography profiles.

Interpretation error is reduced by automated shoreline extraction. This minimises the subjectivity inherent to manual shoreline extraction by a single (or multiple) researcher(s). Of course, it is necessary to recognise that the selection of particular settings when manipulating images and extracting a shoreline involves subjective decision making on behalf of the researcher. Even so, recording these settings enables replicability in a way that tracing does not.

#### RMSV

Selection of the vegetation line as a shoreline proxy is partly an attempt to reduce the error arising from shoreline variability since the vegetation line is less temporally variable than other possible shoreline proxies such as drift or wet/dry lines^35^. In the case of shoreline proxies that record recent water levels, RMSV would be calculated using the variability in water levels of the measured proxy^38^. Given the relative stability of the vegetation line, a dedicated RSMV error calculation is not warranted.

### Elevation change assessment

It is desirable, although not always possible, to quantify the likely elevation deviations from real ground elevations when interpolating and filtering the LiDAR point clouds. It is also pertinent to focus on the specific elevation band that is of interest and to cover a range of elevations when checking for accuracy against ground truth data. The LiDAR from 28^th^ January 2013 is available as a point cloud while that from 28^th^ February 2014 is a pre-processed DEM at 1 m resolution. EA cross-shore topography surveys are available for 8^th^ March 2013 as well as 3^rd^ March 2014, with a further RTK (real time kinematic) field survey carried out by the authors on 31^st^ January 2014 where the intention was to map the geomorphological effects of the 5 December 2013 UK east coast storm surge. Thus, these datasets can be used to assess the accuracy of the LiDAR-derived DEMs post-processing. While it is acknowledged that there is a short time lag between the capture of the LiDAR and the cross-shore topography survey dates which may account for some variation in elevation, these ground truth data allow 1 m LiDAR product accuracy to be evaluated.

**Fig. 6:**
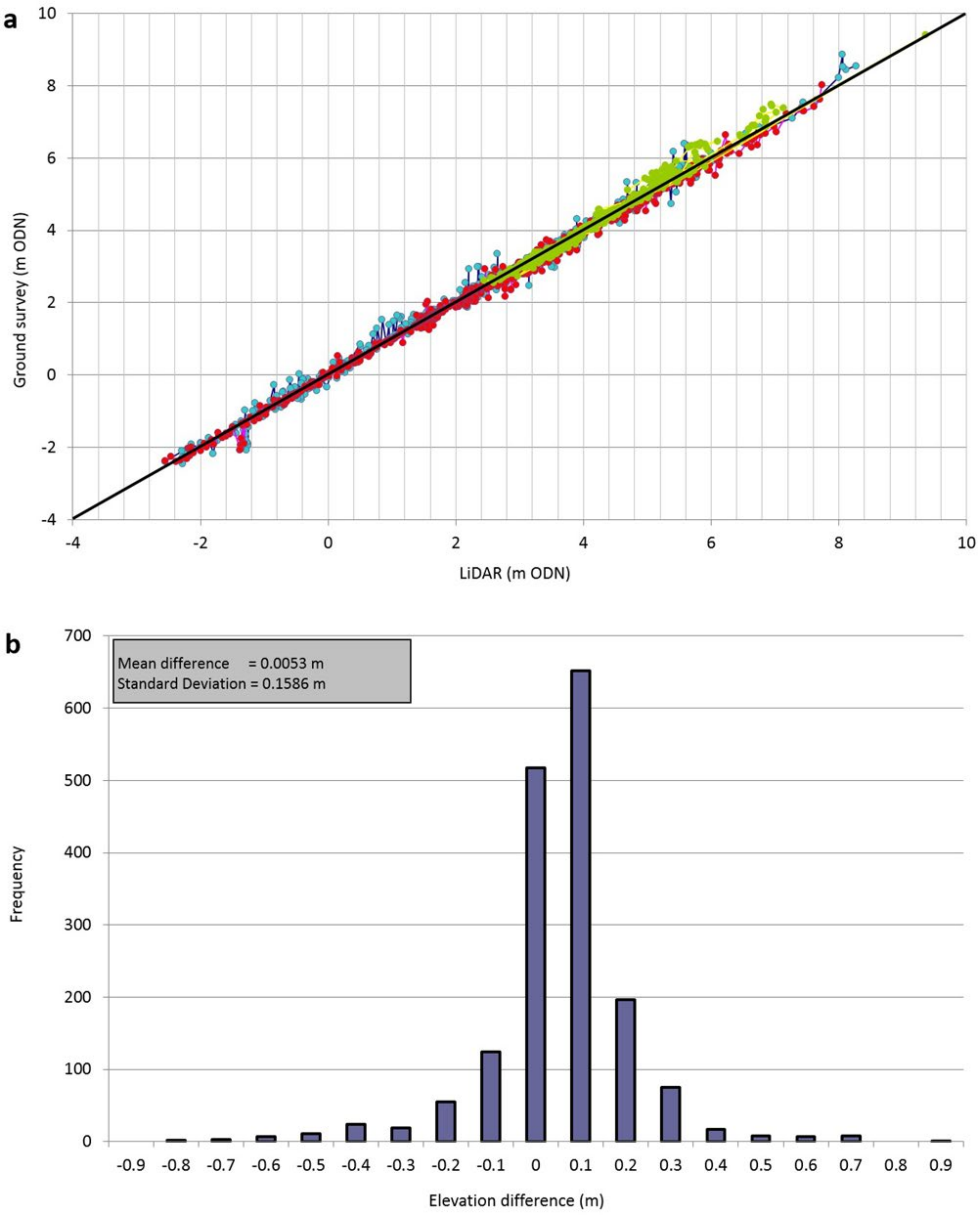
Validation of LiDAR point cloud using ground elevation surveys. (**a**) Ground surface elevations from RTK surveys and LiDAR for the western end of the barrier at Scolt Head Island. 28^th^ January 2013 LiDAR elevations are plotted against EA cross-shore surveys on 8^th^ March (blue); 28th February 2014 LiDAR elevations are plotted against EA cross-shore surveys on 3^rd^ March (red) and ground survey on 31^st^ January (green) for the eroded edge of the shoreline barrier. The 1:1 line is shown in black. In all cases r^2^ > 0.98. (**b**) Frequency distribution plot of mean error calculated in 6a.

The procedure to cross-reference the LiDAR with the cross-shore topography survey data firstly involved the creation of point shapefiles for 6 cross-shore profiles (Data workflow 5). For this case, we selected profiles available within the western Scolt Head Island polygon shapefile including N014 (Figs 1 and 3), giving a total of 603 points. The attribute table contains the point ID, its easting and northing and its elevation. The elevation of the LiDAR-derived raster was extracted at the same location. These two sets of elevations were exported for subsequent Ordinary Least Squares Regression analysis (Fig. 6a). This was performed separately for the 2013 and 2014 cross-shore profiles and for the additional ground survey on 31^st^ January 2014. The mean error between the ground survey and LiDAR DEM of 0.0053 m (0.53 cm) was calculated and the frequency distribution of this mean error is presented in Fig. 6b.

## Usage Notes

These usage notes are intended to guide researchers in executing the procedures that comprise the methodology presented in the paper. The usage notes refer to ArcGIS software to maintain consistency with the datasets deposited in the PANGAEA database^30,31,34,36^.

### Data workflow 1: geo-referencing

The ArcMap tools required for this data workflow include: Geo-Referencing Toolbar.

Data workflow 1 can be executed as follows:

1. Set coordinate system: Start ArcMap and, before you add any data, set a coordinate system for the map. For example: Right click on map background > Data Frame Properties > Coordinate System > Select a coordinate system > Predefined > Projected Coordinate Systems > National Grids > Europe > British National Grid > OK.
2. Add data: Add data > Navigate to folder > Connect to Folder if necessary > Select the dataset > Add > Agree to Create Pyramids if necessary > Acknowledge unknown spatial reference warning if necessary.
3. Mosaic tiles: Navigate to: Data Management tools > Raster > Raster Processing > Mosaic to New Raster. Highlight the tiles you want to mosaic in the Table of Contents and drag them into the ‘Input files’ box. Select a suitable output location and name, e.g. AER_2013_mos.tif. You must include the .tif extension. Select ‘32-bit signed’. Select the appropriate number of bands – for greyscale images, it will be ‘1’, for RGB it will be ‘3’ and for RGBI it will be ‘4’. You can find out the number of bands by clicking on one of the aerial tiles and selecting ‘Properties’. All other parameters should be left as defaults.
4. Load geo-referencing tools: Customize > Toolbars > Geo-referencing. On the Geo-referencing Toolbar, select the layer that requires geo-referencing using the drop-down. If the layer does not share a common projection with the Data Frame, you will not be able to perform the geo-referencing.
5. Select control points: In the Geo-referencing Toolbar > Add Control Points (CPs) > Click the Link Table to open, this will display the CPs you select. CPs should be located close to the feature of interest, and evenly spread across the image. Immobile artificial structures are often appropriate, buildings and boundaries for example.
6. Plot control point: Zoom into map as closely as possible > Triple-click on your chosen CP > An entry will be created in the Link Table with X and Y ‘Source’ coordinates and X and Y ‘Map’ coordinates. The ‘Source’ coordinates refer to the pixel location, do not change these.
7. Enter true values: You must change the ‘Map’ coordinates to the reference CPs, obtained from field surveys or from a map that has been geo-referenced to a high standard. Do this by manually editing the Link Table. Ensure the units are in metres.
8. Plot more control points: Add more CPs; four points are required for a first order or ‘affine’ transformation; five or more points are required to be able to model errors in the CP locations (indicated by the residuals) and overall transformation errors.
9. Perform transformation: Geo-referencing Toolbar > Geo-referencing > Rectify.
10. Save: Save the geo-rectified raster. Leave cell size as is. Leave resampling size as ‘nearest neighbour’. Leave format as TIFF. Name appropriately, e.g. AER_2014_geo.

### Data workflow 2: shoreline extraction from vertical aerial photography

The ArcMap tools required for this data workflow include: ArcMap ArcScan Toolbar; ArcMap Draw Toolbar; ArcMap Raster Calculator; ArcMap Image Analysis; ArcMap Editor Toolbar.

Data workflow 2 can be executed as follows:

1. Set coordinate system: Start ArcMap and, before you add any data, set a coordinate system for the map. For example: Right click on map background > Data Frame Properties > Coordinate System > Select a coordinate system > Predefined > Projected Coordinate Systems > National Grids > Europe > British National Grid > OK.
2. Add data: Add the mosaiced, georeferenced vertical aerial imagery. Add data > Navigate to folder > Connect to Folder if necessary > Select the dataset > Add > Agree to Create Pyramids if necessary > Acknowledge unknown spatial reference warning if necessary.
3. Clip to shoreline zone: To improve the quality and speed of subsequent edge detection processing, it is advisable to clip the mosaiced raster to a smaller, and more specific ‘shoreline zone’. First activate the ‘Draw’ Toolbar: Customize > Toolbars > Draw. Then draw a polygon around the desired area. Then right click on the mosaiced raster in the table of contents > Export data > Select a suitable output location and name, e.g. ‘AER_2013_clip’. Accept the other defaults. Delete the original polygon.
4. Apply edge detection algorithm: On the main ribbon, Navigate to: Windows > Image Analysis > Select the clipped layer in the Image Analysis window > Go to Add Function > This will open the Function Template Editor > Select the layer and right click > Insert Function > Convolution function > Sobel horizontal. This will create a new raster in the Table of Contents. You may wish to rename it, e.g. AER_13_sobh. Starting with the clipped shoreline again, repeat the above but apply the Sobel vertical function. The two Sobels should then be combined using the Raster Calculator: SquareRoot(Square(“AER_13_sobh”) + (Square(“AER_13_sobv”)). Select a suitable output location and name, e.g. ‘AER_13_sobhv’.
5. Convert to bitonal image: This can be done using the Raster Calculator: Spatial Analyst > Map Algebra > Raster Calculator > Con(“layer name” > 25,1,0). Select a suitable output location and name, e.g. ‘AER_2013_bit25’. Choice of an appropriate threshold will depend on the raster in question. One option is to create numerous bitonal images, with varying thresholds. It is easy to visualise the outputs of the vectorisation using the ArcScan Toolbar on multiple different bitonal images.
6. Raster Clean-up: Create a New Shapefile > Type: Polyline > Name appropriately, e.g. AER_2013_vectors. Enter a new edit session in the Editor Toolbar and select the new shapefile. Open the ArcScan Toolbar > Select the appropriate layer to vectorise in the dropdown. First, clean-up the bitonal image. This can be achieved using the Raster Clean-up option provided in the ArcScan Toolbar. Select ‘Start Clean-up’ > then apply the erode, dilation, opening and closing functions to iteratively emphasise the shoreline.
7. Vectorization: Although the settings may have to be altered on a raster-by-raster basis, the choices presented below should provide a useful starting point. Once you have refined your settings, select ‘Vectorise’ and ensure that the newly created shapefile (AER_2013_vectors) is selected as the Template.
  **Vectorization** > **Options**:
  Vectorization method: centreline
  Select appropriate foreground colour
  Advanced > Limit the number of vertices in a polygon to: 0 (i.e. no polygons)
  **Vectorization** > **Vectorization settings**:
  Intersection solution: Geometrical (when secondary lines intersect the main shoreline, they will not skew the main shoreline)
  Max line width: 20
  Noise level: 95%
  Compression tolerance: 0.025
  Smoothing weight: 1
  Gap closure tolerance: disabled
  Hole size: depends on raster (if there are ‘holes’ then enable this option
  Resolve Corners: disabled.
8. Manual edit: The final step is to ensure that the vector generated by ArcScan produces a single continuous shoreline. This requires the vector to be edited using the Editor Toolbar. Once a single line has been tidied up, highlight it and delete all other lines by using the Attribute Table and Editor Toolbar. Select a suitable output location and name, e.g. ‘AER_2013_sline’. Once satisfied, use the Editor Toolbar > Stop Editing > Save Edits.

### Data workflow 3: DEM creation from LiDAR point cloud

The ArcMap tools required for this data workflow include: Laszip; ArcMap Spatial Analyst Tool.

Data workflow 3 can be executed as follows:

1. Set coordinate system: Start ArcMap and, before you add any data, set a coordinate system for the map. For example: Right click on map background > Data Frame Properties > Coordinate System > Select a coordinate system > Predefined > Projected Coordinate Systems > National Grids > Europe > British National Grid > OK.
2. Add LAStools to the ArcToolbox: The downloaded LiDAR point cloud is delivered as a .laz file that needs to be in .las format. Open ArcMap and ArcToolbox. Right click on the ArcToolbox. Add toolbox. Navigate to the folder with the las tools and click on lastools.tbx. This will add the LAStools to the ArcToolbox. A range of python scripts is added, including the script laszip.
3. Run conversion script: Double click the laszip > Navigate to the folder where the .laz point cloud is stored > Select a folder path and filename for the new .las file > Click OK. The conversion should run quickly.
4. Create LAS dataset: Navigate to ArcToolbox LAS dataset > Create LAS dataset > A point cloud will appear on your map. If you are zoomed out too far a red bounding box will appear to show where the point cloud is located. Add as many point cloud .las files as you want to cover your area.
5. Apply ground filter: Select the .las files for your area of interest > Load only these as a single LAS dataset > Provide a sensible folder pathway and filename. Add a ground filter using the LAS Dataset filter facility in the upper left part of the ArcMap toolbar.
6. Convert LAS dataset to raster: ArcToolbox > Conversion Tools > To Raster > LAS Dataset to Raster (Parameters: average cell assignment type; nearest neighbour void fill; cell resolution of 1 m, or whatever resolution you require depending on computational efficiency). Name the raster appropriately, e.g. LID_2013_raster.
7. Select appropriate display Symbology: The DEM will display as a greyscale image. The Symbology can then be adjusted for Elevation Bands and Colour. Appropriate selection of the number of elevation bins will depend on the vertical scale of the dataset. Here, thirty elevation bins were chosen, with those below 0 m ODN having a 0.25 m band, while those above 0 m ODN had a 0.5 m band.
8. Contour raster: The raster can be contoured using Spatial Analyst Tool > Surface > Contour. The contour selected will depend on the processes of interest. MHWS (Mean High Water Springs) is often useful for coastal analysis as is MSL (Mean Sea Level). Name appropriately, e.g. MHWS_2013.

### Data workflow 4: compute DEM of difference (DoD)

The ArcMap tools required for this data workflow include: ArcMap Raster Calculator; ArcMap Editor Toolbar.

Data workflow 4 can be executed as follows:

1. Set coordinate system: Start ArcMap and, before you add any data, set a coordinate system for the map. For example: Right click on map background > Data Frame Properties > Coordinate System > ‘Select a coordinate system’ > Predefined > Projected Coordinate Systems > National Grids > Europe > British National Grid > OK.
2. Add DEMs: Add data > Navigate to folder > Select and add > Agree to Create Pyramids > Acknowledge unknown spatial reference warning.
3. Mosaic: Toolbox > Data Management > Raster > Raster Dataset > Mosaic to New Raster. The settings below provide a starting point. Once settings have been selected, ensure that the rasters are named appropriately, e.g. LID_2013_mos; LID_2014_mos.
  Input: Lidar .asc files
  Output: choose appropriate folder
  Pixel Type (optional): Leave as default. If you have to choose, it is ’32-BIT Signed’
  Number of bands: 1 (This can be found by right-clicking on your layer > Properties > General).
  Mosaic Operator: LAST
  All other settings remain at default.
4. Convert to metres: This step is necessary so you can draw your contour in metres and to ensure both point cloud generated rasters and ASCII rasters are in the same units. Toolbox > Spatial Analyst > Map Algebra > Raster Calculator > Float(“LID_2013_mos.asc”)/1000. Rename the raster appropriately, e.g.: LID_2013_raster.
5. Select appropriate display Symbology: The DEM will display as a greyscale image. The Symbology can then be adjusted for Elevation Bands and Colour. Appropriate selection of the number of elevation bins will depend on the vertical scale of the dataset.
6. Mask region of interest: Create a shapefile covering the area of interest. Right-click on the folder where you want to create the shapefiles, then Select > New > Shapefile > Select Polygon. Use the shapefile to mask an identical area from both DEMs: Spatial Analyst Tools > Extraction > Extract by Mask. This is shown on Fig. 1. The output is two identical spatially referenced rasters in the same units (m). Rename the raster appropriately, e.g.: LID_2013_raster.
7. Calculate difference between DEMs: Use the Minus Tool to take the earlier DEM away from the later one. The output raster should be named appropriately, e.g.: LID_2013_14_dod.

### Data workflow 5: extract topography profiles from LiDAR

The ArcMap tools required for this data workflow include: ArcMap Editor Toolbar; 3D Analyst Tools.

Data workflow 5 can be executed as follows:

1. Create line shapefile: First, create two shapefiles – one polyline-type (which is the template) and one point-type where the points will be created. Right-click on the folder where you want to create the shapefiles, then Select > New > Shapefile. Call the shapefile an appropriate name (e.g. LID_2014_N014s_line), and select the ‘Polyline’ type. In the Editor Toolbar, enter an edit session, selecting the newly created polyline shapefile. Select Create Features > Polyline and draw the line where you want the transect to be. Click on the editor dropdown again > Stop Editing > Save Edits.
2. Create point shapefile: Return to the folder where you just created the polyline dataset. New > Shapefile and create a point shapefile, with an appropriate name, for example, LID_2014_N014s_prof making sure to set ‘Type’ as ‘point’.
3. Create multipoint transect: Return to the Editor Toolbar > Start Editing > Select the polyline shapefile ‘LID_2014_N014s_prof’. Use the black arrow on the Editor Toolbar and select the LID_2014_N014s_line shapefile. Click on the Editor Toolbar dropdown and select Construct Points. This will open a window which allows you to specify the details of the point creation. Ensure the point shapefile that you are currently creating is selected as Template. Under ‘Distance’ enter 0.5. This will create a point at 0.5 m spacing along the line. Click Ok. The minimum spacing is limited by the resolution of the LiDAR dataset. In this case, 0.5 m spacing is appropriate since the final error bound will be 0.5 m either side of the shoreline – or 1 m total, matching the 1 m LiDAR resolution. Click on the editor dropdown again > Stop Editing > Save Edits.
4. Map elevation data: Map the Z values from the LiDAR dataset. This is achieved manually using 3D Analyst Tools > Functional Surface > Add Surface Information. The Input Feature Class is the point type shapefile. Input Surface is the LiDAR dataset. In the Output Property box, ensure that ‘Z’ is ticked. Leave all other options as default > Click Ok. If you are using multiple overlapping LiDAR datasets, Add Surface Information must be performed multiple times, mapping on elevation values from coarsest to finest. In the shapefile Attribute Table, a Z column appears. The output is LID_2014_N014s_prof.

## ISA-Tab metadata file

Download metadata file

## Data Availability

The datasets included in this paper were manipulated using ESRI ArcGIS v10.2 and later versions. All the ArcMap Tools referred to in the Data Usage section are available in version 10.2 up to the current version, 10.6. The digital shoreline analysis was performed using the open access R-package, AMBUR^39^. LiDAR point cloud manipulation was performed using the open access software, lasizp (https://laszip.org/).
